# Supplementary material for: Algorithm guided outlining of 105 pancreatic cancer liver metastases in Ultrasound
Source: Sci Rep. 2017 Oct 6;7:12779. doi: 10.1038/s41598-017-12925-z (PMC5630585; doi:10.1038/s41598-017-12925-z)
Supplement: Supplementary file 1 — Supplementary Table 1 and Supplementary Figures 1 - 5 [file 41598_2017_12925_MOESM1_ESM.doc]

**Title**

Algorithm guided outlining of 105 pancreatic cancer liver metastases in Ultrasound

**Authors**

Alexander Hann^a,b^,*, Lucas Bettac^a^, Mark M. Haenle^a^, Tilmann Graeter^c^, Andreas Berger^a^, Jens Dreyhaupt^d^, Dieter Schmalstieg^e^, Wolfram G. Zoller^b^, Jan Egger^e,f^

*^a^ Department of Internal Medicine I, Ulm University, Ulm, Germany*

^b^ Department of Internal Medicine and Gastroenterology, Katharinenhospital, Kriegsbergstraße 60, 70174 Stuttgart, Germany.

^c^ *Department of Diagnostic and Interventional Radiology, Ulm University, Ulm, Germany.*

*^d^ Institute of Epidemiology & Medical Biometry, Ulm University, Ulm, Germany.*

^e^ Institute for Computer Graphics and Vision, Graz University of Technology, Inffeldgasse 16, 8010 Graz, Austria.

^f^ BioTechMed, Krenngasse 37/1, 8010 Graz, Austria.

* Corresponding author: alexander.hann@uniklinik-ulm.de

**Corresponding author:**

Dr. med. Alexander Hann

Uniklinik Ulm

Klinik für Innere Medizin I

Albert-Einstein-Allee 23

89081 Ulm

Email: [alexander.hann@uniklinik-ulm.de](mailto:alexander.hann@uniklinik-ulm.de)

Tel.: 0049 731 / 500 44750

Fax: 0049 731 / 500 44502

**Supplementary Tables:**

|  | **Difference Diameter a (mm)** | | | | | **Difference Diameter b (mm)** | | | | |
| --- | --- | --- | --- | --- | --- | --- | --- | --- | --- | --- |
|  | **Median** | **Q_1_** | **Q_3_** | **Min** | **Max** | **Median** | **Q_1_** | **Q_3_** | **Min** | **Max** |
| Examiner 1 (n=92) | 2 | 1 | 4 | 0 | 20 | 1 | 0 | 2 | 0 | 11 |
| Examiner 2 (n=94) | 3 | 2 | 5 | 0 | 20 | 2 | 1 | 3 | 0 | 11 |

**Supplementary Table 1:** Comparison of the difference of maximal manual and semiautomatic segmented diameter per examiner. Q_1_ and Q_3_ = quartile 1 and 3.

**Supplementary Figures:**


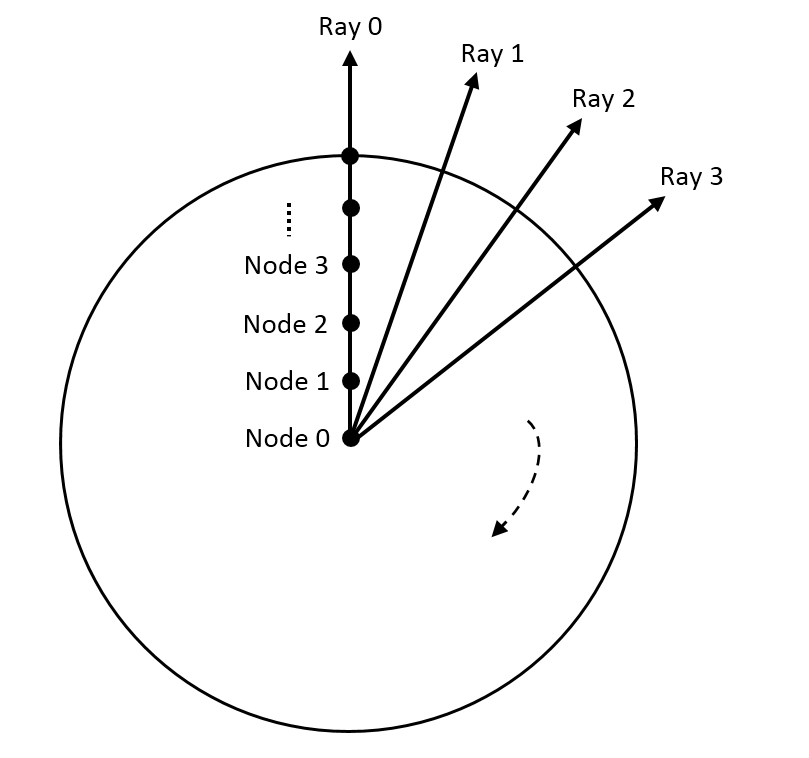


**Supplementary Figure 1:** Graphs’ nodes sampled along radial rays, which are equidistantly distributed around a fixed point in a clockwise manner.


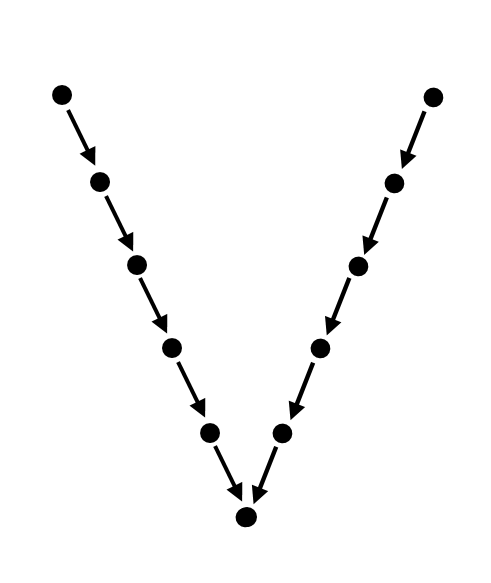


**Supplementary Figure 2:** Intra-edges that connect nodes along the same ray.


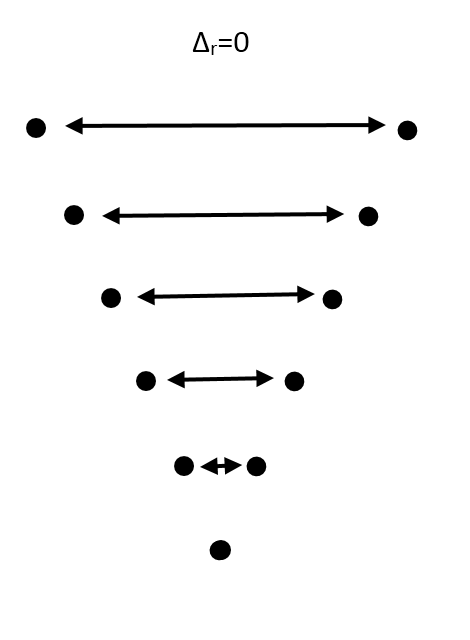


**Supplementary Figure 3:** Inter-edges between nodes from different rays with a delta value of zero.


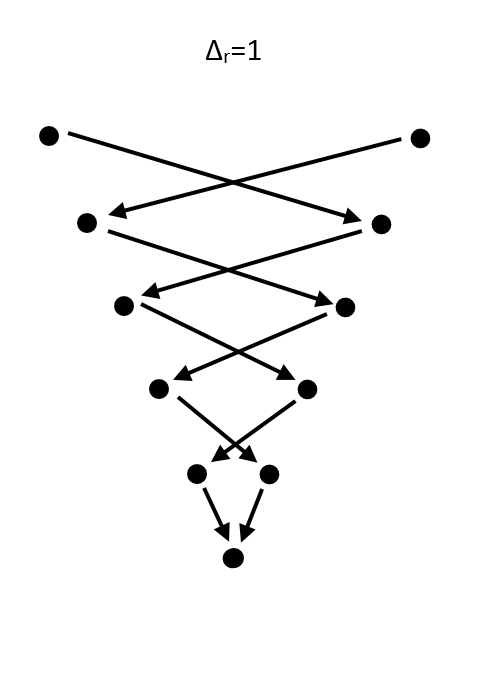


**Supplementary Figure 4:** Inter-edges between nodes from different rays with a delta value of one.


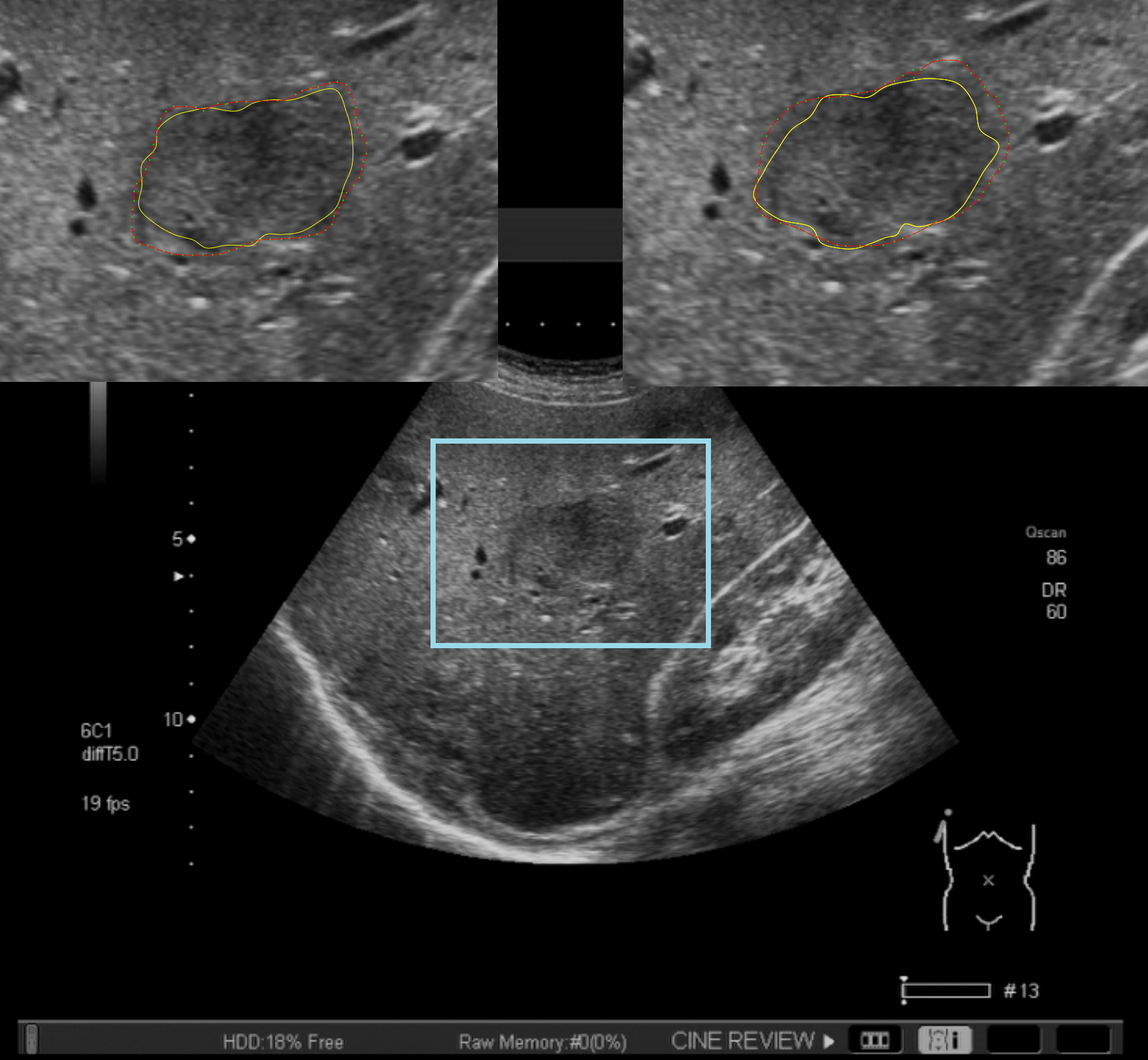


**Supplementary Figure 5:** Example of a segmentation marked as inadequate by examiner 1. Depicted are the native image in the background with two zoomed view of the metastasis (representing the blue box). The upper left zoomed view represents the segmentation results of examiner 1 and the upper right box the results of examiner 2. The red outlines represent the manual segmentations and the yellow outlines represent the results of the semi-automatic segmentation.
